# Environmental Impacts, Land‐Use Trade‐Offs, and Sustainable Management Pathways of *Eucalyptus* Plantation Expansion in Ethiopia

**DOI:** 10.1155/tswj/6629432

**Published:** 2026-02-24

**Authors:** Yohannes Gelaye, Kiros Getachew

**Affiliations:** ^1^ Department of Horticulture, College of Agriculture and Natural Resources, Debre Markos University, Debre Markos, Amhara, Ethiopia, dmu.edu.et; ^2^ Department of Natural Resources Management, College of Agriculture and Natural Resources, Debre Markos University, Debre Markos, Amhara, Ethiopia, dmu.edu.et

**Keywords:** deforestation, environmental impacts, *Eucalyptus* plantation, indigenous tree species

## Abstract

**Purpose:**

*Eucalyptus* plantations have increasingly transformed the Ethiopian landscape due to their high economic returns from timber, fuelwood, and household income generation. Their rapid expansion has become particularly prominent in smallholder farming systems and periurban areas. Despite these socioeconomic benefits, *Eucalyptus* plantations have raised serious environmental concerns, especially in water‐scarce and biodiversity‐rich regions. A comprehensive evaluation of their environmental trade‐offs is therefore essential to support evidence‐based and sustainable land‐use planning.

**Method:**

This review synthesizes findings from peer‐reviewed literature and empirical studies on *Eucalyptus* cultivation in Ethiopia. The analysis focuses on documented impacts on water resources, soil properties, biodiversity, and ecosystem functioning while also assessing reported mitigation practices.

**Results:**

The review reveals that *Eucalyptus* plantations exhibit high water consumption, often exacerbating local water scarcity and competing with adjacent agricultural crops. Soil nutrient depletion, increased erosion, and reduced understory vegetation are frequently reported, alongside declines in native plant and faunal diversity. Allelopathic effects further limit the regeneration of indigenous species.

**Discussion:**

Although environmental drawbacks are evident, *Eucalyptus* plantations contribute to carbon sequestration, fuelwood supply, and rural livelihoods. These benefits indicate the need for improved management rather than complete exclusion of *Eucalyptus* from farming landscapes.

**Conclusion:**

Sustainable management approaches, particularly agroforestry and mixed‐species systems, can reduce negative impacts while maintaining socioeconomic benefits. Integrating *Eucalyptus* with indigenous and multipurpose species such as *Cordia africana*, *Faidherbia albida*, and *Acacia abyssinica* is recommended to enhance soil fertility, maintain biodiversity, and promote ecosystem resilience.

## 1. Introduction

In Ethiopia, approximately 80,000 ha of natural forest have been transformed into agricultural land. Of the total woodland areas affected, an estimated 62.5% have been cleared primarily for charcoal production, while the remaining 37.5% of bushland has been removed to meet the demand for fuelwood. This extensive deforestation reflects a significant shift in land use driven by energy needs and subsistence agriculture, contributing to ongoing environmental degradation and loss of biodiversity [1]. The rapid population growth in Ethiopia has spurred a heightened demand for wood industries, construction, and fuel, driving the planting of fast‐growing, multipurpose exotic tree species as a solution [2].

In the early 1980s, Ethiopia initiated the introduction of fast‐growing *Eucalyptus* species as part of its afforestation and reforestation efforts. Since then, *Eucalyptus* has emerged as one of the most extensively cultivated tree species in the country, currently covering an estimated 506,000 ha of land. Its widespread adoption underscores its economic and ecological significance, particularly in addressing wood supply shortages and land rehabilitation challenges [3]. Between 1978 and 2020, a total of 133,041 ha of community‐based tree plantations were established across Ethiopia. Of this total, *Eucalyptus* species account for approximately 58%, highlighting their dominant role in community forestry initiatives and their continued importance in meeting local wood demand and supporting rural livelihoods [4]. Despite being annually cultivated on fertile lands for their economic benefits, *Eucalyptus* trees have been associated with a range of negative environmental consequences. Farmers in the Amhara Region have particularly reported concerns such as soil degradation and increased water scarcity, underscoring the ecological trade‐offs linked to the widespread expansion of this species [5]. In Ethiopia, the literature has largely concentrated on the socioeconomic dimensions of *Eucalyptus* plantations, while environmental impacts have received comparatively limited attention [6].

These environmental impacts are highly context‐specific and vary across agroecological zones, plantation management practices, and landscape settings, making generalized conclusions difficult. Moreover, fragmented and sometimes contradictory findings across studies have contributed to ongoing debates regarding the ecological sustainability of *Eucalyptus* plantations in Ethiopia. The absence of an integrated synthesis that systematically evaluates water, soil, biodiversity, and land‐use trade‐offs has limited the formulation of coherent management and policy responses [7]. Addressing these gaps is essential for informing evidence‐based land‐use planning and promoting environmentally sustainable plantation practices. Accordingly, this review seeks to examine the expansion of *Eucalyptus* plantations in Ethiopia and to critically assess their associated environmental implications.

## 2. Literature Review

### 2.1. Origin and Nature of the *Eucalyptus* Tree

*Eucalyptus* is a widely recognized evergreen tree species belonging to the family Myrtaceae and is native to regions including Australia, Indonesia, and the Philippines. It is distinguished by its unique biological and ecological characteristics, which contribute to its broad adaptability and diverse applications [8]. With over 900 *Eucalyptus* species, approximately 100 are considered economically significant, representing a select group with notable economic value [9].

First introduced to Portugal approximately four centuries ago, the widely cultivated *Eucalyptus* tree has since gained global prominence due to its diverse benefits. It serves as a valuable source of timber, paper pulp, medicinal and essential oils, and fuelwood. In addition, it provides ecological functions such as offering shade and acting as an effective windbreak, thereby contributing significantly to both industrial sectors and environmental systems [10].

The term *Eucalyptus* was first introduced in 1799 by French botanist Jacques‐Julien Houtou de La Billardière during his taxonomic classification of the genus. The name is derived from the Greek words “eu” (meaning “well”) and “kalyptos” (meaning “covered” or “hidden”), in reference to the operculum that conceals the flower buds of the species [11]. *Eucalyptus* trees are characterized by their tall, evergreen stature, often reaching heights of up to 60 m. They are well adapted to thrive at elevations exceeding 1850 m above sea level [12].

### 2.2. History and Trends of *Eucalyptus* Plantation in Ethiopia

*Eucalyptus* was introduced to East Africa in the 19^th^ and 20^th^ centuries by Europeans to address forest degradation and wood shortages [6]. Belgian missionaries brought it to Rwanda in the early 1900s to meet the growing demand for fuel and construction wood [13].

In 1894/95, Emperor Menelik II′s advisors introduced *Eucalyptus* to Ethiopia to provide fuel wood and construction timber for Addis Ababa′s growth. The fast growth and adaptability of *Eucalyptus* made it a widely planted species in Ethiopia, with people quickly planting it around their homes in Addis Ababa [14]. Between the early 1970s and 1994, *Eucalyptus* plantations expanded to cover approximately 15,000 ha near Addis Ababa and an additional 76,000 ha across various regions of Ethiopia. This expansion was largely facilitated by the support of international donors, which played a crucial role in promoting the development of new rural plantations [13]. At present, Ethiopia cultivates a total of 55 *Eucalyptus* species, of which approximately 5–10 are widely planted on a large scale. Among the most commonly cultivated species are *Eucalyptus globulus* and *Eucalyptus camaldulensis*, which are predominantly grown in the highland regions for various wood‐based purposes, including fuelwood, construction, and timber production [15]. In Northwestern Ethiopia, farmers increasingly established fast‐growing *Eucalyptus* trees in woodlots, resulting in these species accounting for 58% of all planted forests by 2010. This trend reflects a significant rise in the annual planting rate of *Eucalyptus*, driven by its rapid growth and economic viability [16].

### 2.3. The Expansion of *Eucalyptus* Plantation in Ethiopia

The transformation of forests and marginal lands into agricultural areas, driven by population growth and unsustainable resource management practices, has resulted in wood shortages and subsequently stimulated the expansion of *Eucalyptus* plantations throughout the highland regions of Ethiopia [17]. These plantations, which are prized for their productivity and economy, require careful monitoring due to environmental risks and impacts on adjacent crops [18].

Despite farmers′ awareness, the demand and ease of growing *Eucalyptus* trees led to rapid plantation expansion in Ethiopia, driven by rising wood prices since the 19^th^ century [19]. Furthermore, the ease of growing and high productivity of *Eucalyptus* trees have also facilitated their plantation expansion.

Between the 1980s and 2010, *Eucalyptus* plantations experienced rapid expansion, driven largely by households annually cultivating more than 100 species of red or white *Eucalyptus* trees. This sustained planting activity contributed to a continuous increase in the overall plantation area during this period [14]. A 2023 survey reported that *Eucalyptus* plantations in Ethiopia′s Oromia, Amhara, and Southern Nations, Nationalities, and Peoples′ regions collectively covered approximately 68,000 ha. Among these, Oromia accounted for 43.68% of the total area, Amhara comprised 26.47%, and the Southern Nations, Nationalities, and Peoples′ region represented 29.85% [20].

A 2015 study conducted across three regions in Ethiopia revealed that *Eucalyptus* accounted for the largest proportion of plantation area at 45%, followed by *Cypress* at 42%, *Juniperus* at 3%, *Pine* at 2%, and *Grevillea* at 1%, with other species collectively comprising 7% of the total plantation coverage (Table 1).

**Table 1:** Spatial extent of *Eucalyptus* plantation in relation to other industrial plantations in three Ethiopian regions. Reproduced from Bekele [14].

No.	Tree species	*Eucalyptus* and other tree species coverage (%)
1	*Eucalyptus*	45
2	*Cypress*	42
3	*Juniperus*	3
4	*Pine*	2
5	*Grevillea*	1
6	Others	7
	Total	45

### 2.4. Geographical Distribution of *Eucalyptus* in the World and East Africa

**Figure 1:**
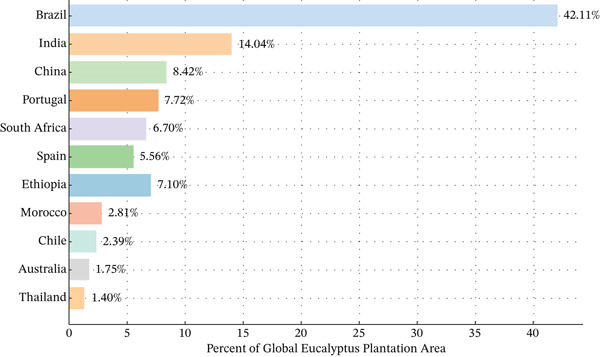
*Eucalyptus* plantation area coverage by country. *Source:* [22].

*Eucalyptus* is considered one of the most widely distributed tree species globally, with its cultivation spanning more than seven billion hectares worldwide [21]. According to a study conducted in 2000, Brazil ranked first in terms of *Eucalyptus* plantation area, accounting for 42.11% of the global total, while Ethiopia held the fifth position, comprising 7.10% of the total plantation coverage (Figure 1).

### 2.5. Role of the *Eucalyptus* Tree in Ethiopia

#### 2.5.1. Socioeconomic Roles of *Eucalyptus* Tree in Ethiopia

Changes in land use are predominantly driven by a combination of socioeconomic, environmental, and cultural factors, alongside local livelihoods, governance policies, regulatory frameworks, and prevailing cultural norms [23]. In Southern Ethiopia, the transition toward monoculture cultivation has been primarily motivated by the economic advantages associated with crops such as chat (*Catha edulis*) and *Eucalyptus* [24]. Farmers are progressively cultivating *Eucalyptus* trees as a means to enhance their household income, with this crop potentially contributing up to 50% of their total earnings [25].

*Eucalyptus* is extensively cultivated owing to its capacity to meet the increasing demand for fuelwood and construction materials, in addition to its wide range of other practical applications [15]. For example, in south‐central Ethiopia, *Eucalyptus* trees provide over 100% of the construction wood, 20% of the charcoal, and 93% of other wood products [26]. A comparable study conducted in the Lake Plain region revealed that *Eucalyptus* was primarily utilized for fuelwood, income generation, and construction purposes, with little to no attention given to environmental conservation considerations [27]. *Eucalyptus* also possesses medicinal benefits, with applications in the treatment of common colds, flu, and fever [28]. Numerous studies conducted in Ethiopia have reported the substantial economic significance of the *Eucalyptus* tree, particularly in relation to rural livelihoods and local market dynamics [29].

Although Ethiopian farmers often prioritize the cultivation of *Eucalyptus* over cereal crops due to its superior income‐generating potential, its adverse environmental impacts are frequently overlooked. The economic returns from *Eucalyptus* are relied upon to support household income and food security, particularly in areas facing challenging agroecological conditions [30].

#### 2.5.2. Environmental Roles of the *Eucalyptus* Tree in Ethiopia

The introduction of *Eucalyptus* trees in Ethiopia has played a pivotal role in reducing pressure on natural forests by helping to meet the growing demand for firewood and construction materials [15]. *Eucalyptus* trees′ rapid growth and ability to store large amounts of biomass effectively contribute to carbon sequestration, supporting efforts to address global warming [31]. Besides, *Eucalyptus*′ rapid growth and high‐quality wood have resulted in a reduction in deforestation, thus preserving biodiversity and indigenous species, providing environmental benefits [32]. Despite these benefits, *Eucalyptus* trees have faced criticism for inhibiting the growth of underground plants through shading and decreasing crop yields in areas near plantations [33].

### 2.6. The Causes of *Eucalyptus* Plantation Expansion in Ethiopia

In the Eza Woreda District of Ethiopia′s West Guarage Zone, research has identified socioeconomic and environmental factors as the primary drivers influencing the widespread cultivation of *Eucalyptus* trees throughout the country [34]. In addition, a study conducted in the Lake Tana watershed of Northwestern Ethiopia identified three primary factors driving the expansion of *Eucalyptus* plantations: socioeconomic conditions, the ecological characteristics of the region, and the inherent biological traits of *Eucalyptus* species. These factors are elaborated upon in the following sections [35].

#### 2.6.1. Socioeconomic Factors

**Figure 2:**
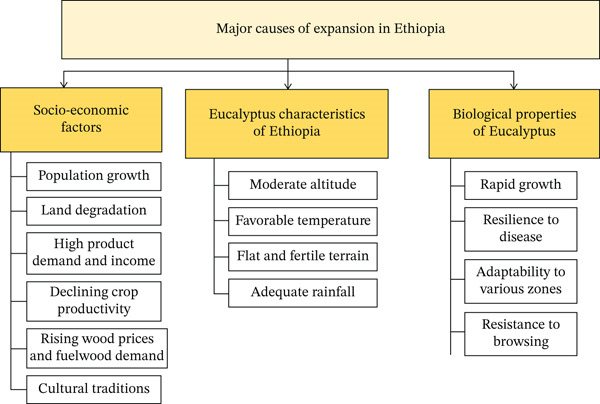
Causes of *Eucalyptus* plantation expansion in Ethiopia.

In Ethiopia′s West Guarage Zone, the cultivation of *Eucalyptus* has markedly increased in response to population growth and land degradation (Figure 2). Farmers plant an average of 61 trees each, contributing to a 70% expansion in plantation area over the past four decades, particularly on uneven and marginal terrains [34].

The expansion of *Eucalyptus* farming is fueled by high product demand and income from woodlot production, with the Amhara Region exporting 3,774,461 poles to Sudan and earning $12,214,327 between 2006/07 and 2010/11 [36].

In northern and south‐central Ethiopia, numerous farmers have transformed their agricultural lands into *Eucalyptus* woodlots. This shift is driven by declining crop productivity, increasing market prices for *Eucalyptus*, the species′ adaptability and rapid growth rate, its resistance to livestock browsing, and the substantial income generated from the sale of wood products [19].

The expansion of *Eucalyptus* plantations in Ethiopia is propelled by several factors, including rising wood prices, superior economic returns, expanding market opportunities, increasing input costs for alternative resources, heightened demand for fuelwood and construction materials, as well as cultural traditions such as Meskel and Arifa, which entail significant wood consumption [37].

#### 2.6.2. The Ecological Characteristics of Ethiopia

While *Eucalyptus* species are adaptable to diverse agroecological zones, the conditions present in Ethiopia, exemplified by the Koga watershed in the western Amhara Region, provide particularly favorable environments. This suitability is attributed to factors such as moderate altitude, optimal temperature ranges, flat terrain, fertile soils, and sufficient rainfall [38].

#### 2.6.3. The Biological Properties of the *Eucalyptus* Tree

*Eucalyptus* is widely valued for its fast growth, hardiness, and resistance to pests and diseases, despite concerns about its potential adverse effects on neighboring crops and the relatively high labor demands it requires. Nevertheless, research indicates that an overwhelming majority of farmers believe its advantages substantially outweigh its drawbacks, and most report a strong intention to continue cultivating *Eucalyptus* in the future [39].

### 2.7. The Impact of *Eucalyptus* Plantation on the Environment in Ethiopia

*Eucalyptus* trees, recognized for their rapid growth and significant carbon sequestration capacity, have generated considerable debate among researchers and stakeholders in Ethiopia [25].

Although some stakeholders oppose the cultivation of *Eucalyptus* trees, others advocate for their cautious use due to the species′ notable benefits. *Eucalyptus* thrives on degraded, swampy, infertile, and arid soils, rendering it an exceptional producer of biomass under challenging environmental conditions [40]. These trees are capable of sequestering more carbon than alternative species, making them beneficial for carbon trading [41].

In the Ethiopian highlands, the annual wood yield of *Eucalyptus globulus* varies between 168 and 2900 kg/ha, contingent upon factors including soil characteristics, stand age, and rotation length [42]. Critics caution that *Eucalyptus* plantations may adversely impact the environment by causing soil nutrient depletion, diminishing water resources, displacing indigenous vegetation, exerting allelopathic effects on surrounding plant growth, and posing risks to ecological stability and biodiversity [43].

#### 2.7.1. Impacts of *Eucalyptus* on Climate

The impact of *Eucalyptus* plantations on the local climate is significant, as their elevated evapotranspiration rates contribute to a reduction in groundwater levels [44]. On average, an individual *Eucalyptus* tree transpires between 20 and 40 L of water daily, resulting in substantial soil moisture depletion within plantations. This process may contribute to desertification and a reduction in local precipitation; however, accurately quantifying its distinct impact on regional climate relative to native forests remains a complex challenge [45].

The introduction of *Eucalyptus* into *Acacia* forest regions has been extensively documented to affect local microclimates. These impacts are influenced by variables such as the basal area of tree cover, which modulates temperature and humidity, as well as leaf size and orientation, which contribute to shading dynamics [46].

#### 2.7.2. Impacts of *Eucalyptus* on Water Resources

*Eucalyptus* plantations face challenges such as decreased water availability and impaired regulation of water flow within steep slope watersheds when compared to natural forests. These effects are influenced by factors including litter deposition, vegetation cover, soil characteristics, and climatic conditions. While these plantations generate greater runoff than grasslands and natural forests, their runoff is comparatively lower than that of cultivated lands [47].

*Eucalyptus* plantations may adversely affect water resources by modifying runoff patterns, diminishing soil moisture, and reducing groundwater recharge. This is exemplified in Ethiopia′s central highlands, where the conversion of cultivated land to *Eucalyptus* plantations has resulted in a 51.1% decline in soil moisture and a 48.9% reduction in spring water flow [48]. In the Koga watershed of Ethiopia′s Amhara Region, the overextraction of groundwater by *Eucalyptus* plantations can cause springs to dry up and alter water flow, leading to a reduction in the groundwater table [38].

In the Lake Tana Basin, *Eucalyptus* plantations have been shown to reduce groundwater availability, causing fluctuations in the water table of up to 3.1 cm daily. Transpiration rates can reach a peak of 1.65 mm/h, while evapotranspiration during dry periods may total 2300 mm, surpassing evapotranspiration levels in non‐*Eucalyptus* areas by approximately 1400 mm [49]. The average daily evapotranspiration rate is nearly double the reference rate and surpasses the actual rate by 2.5 times in fallow agricultural fields [4].

The cultivation of *Eucalyptus* trees may intensify water scarcity due to their higher rate of soil water extraction compared to other tree species [5]. *Eucalyptus* trees are recognized for their contribution to desertification due to their ability to access deep soil moisture and their elevated evapotranspiration rates, which collectively deplete water resources. For example, *Eucalyptus grandis* consumes water at a rate twice that of *Pinus patula* and up to five times more than similarly sized *Podocarpus* and *Cupressus* species during dry seasons [50]. While *Eucalyptus* trees may not markedly exceed other tree species or crops in individual water consumption, their substantial biomass production results in an overall elevated water demand [51]. Thus, the removal of *Eucalyptus* forests can enhance water yield and elevate water tables in downstream regions.

#### 2.7.3. Impacts of *Eucalyptus* on Soil

*Eucalyptus* trees, characterized by their deep root systems and often exacerbated by inadequate forestry management practices, contribute to soil nutrient depletion and diminished agricultural productivity. This phenomenon was substantiated by a social survey that identified a decline in soil fertility attributed to the tree′s extensive nutrient uptake extending beyond the root zones of adjacent crops [49]. Fast‐growing *Eucalyptus* trees with short rotation periods deplete soil nutrients more rapidly compared to slow‐growing species [52]. Upon harvesting, *Eucalyptus* trees extract a substantial quantity of essential macronutrients contained within their aboveground biomass, as they have the capacity to accumulate considerable levels of nitrogen, phosphorus, potassium, calcium, and magnesium from the soil [53]. *Eucalyptus* plantations influence soil moisture content, exhibiting reduced levels in proximity to their stands compared to those near *Croton macrostachyus* during the dry season; however, soil pH, organic matter, exchangeable potassium, and bulk density remain largely unaffected [54]. In the semihumid Ethiopian Highlands on the Lake Tana Plain, research has identified strongly hydrophobic soil conditions in proximity to *Eucalyptus* stands. In addition, variations in macronutrient concentrations generally indicate elevated levels at increasing distances from the *Eucalyptus* plantations [55].

#### 2.7.4. Impacts of *Eucalyptus* on Crop

Owing to their rapid growth and extensive root systems, *Eucalyptus* trees compete with adjacent crops for essential nutrients and water, resulting in the depletion of soil fertility within the vicinity of their plantations by extracting nutrients beyond the root zones accessible to crops [56]. *Eucalyptus* frequently affects adjacent crops through allelopathic interactions, wherein its biologically active compounds modify the chemical environment, thereby inhibiting or stimulating the growth of surrounding organisms. These compounds interfere with critical physiological processes such as cell division, enzyme activity, and photosynthesis, ultimately influencing the development and growth of neighboring plants [57].

Certain species of *Eucalyptus* synthesize chemical compounds in their leaves or litter that suppress the germination and growth of other plant species. Specifically, root extracts of *Eucalyptus camaldulensis* have been shown to negatively affect the germination and early development of certain tree species, while solvent extracts derived from the leaves of *Eucalyptus globulus* inhibit seedling growth and decrease germination rates [57]. In addition, the suppressive impact heightened as the concentration of the extract increased.

The investigation into *Eucalyptus camaldulensis*′ allelopathic impact on tomato crops revealed that inhibition increased with higher extract concentration (5%–10%), displaying the strongest effect, particularly on root length and germination efficiency, posing a potential threat to the vegetable industry in small‐scale farming conditions [58].

The cultivation of *Eucalyptus globulus* poses several challenges, such as competition with cropland (74.5%), shading effects on crops (56.4%), and conflicts over land use due to border effects (27.7%) [48]. The reduction in light intensity is attributed to the dense root network of *Eucalyptus* trees, with observations indicating the presence of approximately 600 roots per square meter within the upper 60 cm of the soil profile at a distance of five meters from the tree [54]. Eucalyptus plantations affect both food security and agrobiodiversity, as evidenced by a tenfold increase in maize biomass and yield observed at distances exceeding 20 m from the plantation boundary [59].

#### 2.7.5. Impacts of *Eucalyptus* on Biodiversity

**Figure 3:**
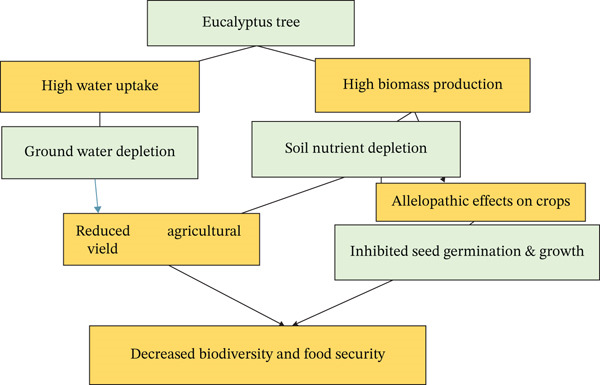
Conceptual model illustrating the ecological impacts of *Eucalyptus* plantations in Ethiopia.

*Eucalyptus* plantations have been shown to suppress underground root development, deplete soil nutrients, and exacerbate soil erosion, thereby diminishing plant biodiversity. Moreover, allelopathic effects associated with *Eucalyptus* inhibit the root elongation of native species and hinder tomato plant growth, contributing to a decline in wetland biodiversity in areas predominantly converted to *Eucalyptus* cultivation, as documented by recent studies [60]. Cultivating tomatoes within a 20‐m radius of *Eucalyptus* plantations is discouraged due to significant yield reductions attributed to soil water repellency, which adversely affects the growth of adjacent plants (Figure 3). To mitigate the negative effects of *Eucalyptus*, the incorporation of nitrogen‐fixing species to establish mixed stands is recommended [58].

### 2.8. Review Gaps and Future Line of Works

The investigation into the expansion of *Eucalyptus* plantations in Ethiopia highlights several gaps and identifies critical avenues for future research concerning their environmental impacts. Comprehensive, long‐term studies are imperative to fully assess the effects of *Eucalyptus* plantations on soil fertility, water resources, and biodiversity within the Ethiopian context. Existing research often exhibits a limited scope, thereby leaving significant knowledge gaps regarding the overall environmental consequences of *Eucalyptus* proliferation. This underscores the necessity for integrated, multidisciplinary approaches in forthcoming studies.

Moreover, it is essential to explore the socioeconomic ramifications of *Eucalyptus* plantation expansion on local communities, particularly smallholder farmers. Although *Eucalyptus* cultivation can provide economic advantages, such as income from timber sales, it may also provoke land‐use conflicts, reduce agricultural productivity, and threaten food security. A nuanced understanding of these interactions is crucial for the formulation of sustainable land management policies.

Finally, future research should prioritize the identification and evaluation of suitable agroforestry practices that integrate *Eucalyptus* with other tree species and crops in Ethiopia. Agroforestry systems present multiple benefits, including improved soil fertility, enhanced biodiversity, and diversified livelihoods for farming communities. Investigating the feasibility and effectiveness of agroforestry models involving *Eucalyptus* will aid in harmonizing economic growth with environmental conservation, thereby advancing sustainable land management strategies in Ethiopia.

## 3. Conclusion and Recommendation

The expansion of *Eucalyptus* plantations in Ethiopia constitutes both opportunities and challenges for sustainable land management. Regardless of the fact that *Eucalyptus* cultivation provides substantial economic benefits, particularly through timber production, fuelwood supply, and rural income generation, it also entails significant environmental risks, including soil nutrient depletion, biodiversity loss, and increased pressure on water resources. These impacts are especially pronounced in ecologically fragile, water‐limited, and densely populated landscapes. Hence, addressing these complex trade‐offs requires an integrated and interdisciplinary approach that combines long‐term environmental monitoring with socioeconomic assessments to better understand the cumulative effects of *Eucalyptus* expansion on ecosystems and smallholder livelihoods.

From an ecological perspective, the adoption of mixed‐species and agroforestry systems is strongly recommended to enhance habitat diversity, improve soil structure, and support native biodiversity. Environmentally, regulating plantation density, site selection, and harvesting intensity is essential to minimize water depletion, soil degradation, and carbon emissions, particularly in sensitive ecosystems. Socioeconomically, promoting diversified livelihood strategies, including the integration of food crops and indigenous multipurpose tree species within *Eucalyptus*‐based systems, can reduce farmers′ dependence on monoculture plantations and enhance income resilience.

Therefore, policymakers and stakeholders should promote sustainable land‐use policies that support mixed‐species agroforestry, invest in long‐term ecological and socioeconomic research, and establish regulatory frameworks for *Eucalyptus* cultivation in vulnerable areas. Lastly, active participation of local communities in land‐use planning and access to technical and financial support will be crucial for balancing economic development with environmental conservation and social well‐being.

## Author Contributions

**Yohannes Gelaye:** conceptualization, data curation, formal analysis, investigation of the study, draft of the original manuscript, visualization, review, editing, approval of the final version of the manuscript. **Kiros Getachew:** revision and editing of the manuscript.

## Funding

No funding was received for this manuscript.

## Disclosure

Both authors have read and approved the final version of the manuscript. Yohannes Gelaye had full access to all of the data synthesis and takes complete responsibility for the integrity of the data and accuracy of the synthesis and analysis.

## Ethics Statement

The authors have nothing to report.

## Consent

The authors have nothing to report.

## Conflicts of Interest

The authors declare no conflicts of interest.

## Data Availability

Data sharing is not applicable to this article, as no new data were created or analyzed in this study.
